# Silencing the Nucleocytoplasmic *O*-GlcNAc Transferase Reduces Proliferation, Adhesion, and Migration of Cancer and Fetal Human Colon Cell Lines

**DOI:** 10.3389/fendo.2016.00046

**Published:** 2016-05-25

**Authors:** Agata Steenackers, Stéphanie Olivier-Van Stichelen, Steffi F. Baldini, Vanessa Dehennaut, Robert-Alain Toillon, Xuefen Le Bourhis, Ikram El Yazidi-Belkoura, Tony Lefebvre

**Affiliations:** ^1^CNRS, UMR 8576, UGSF, Unité de Glycobiologie Structurale et Fonctionnelle, FRABio FR 3688, University of Lille, Lille, France; ^2^Laboratory of Cellular and Molecular Biology, National Institute of Diabetes and Digestive and Kidney Diseases, National Institute of Health, Bethesda, MD, USA; ^3^CNRS, UMR 8161, M3T, Mechanisms of Tumorigenesis and Targeted Therapies, «Institut de Biologie de Lille», Pasteur Institute of Lille, FRABio FR 3688, University of Lille, Lille Cedex, France; ^4^U908, CPAC, Cell Plasticity and Cancer, INSERM, University of Lille, Lille, France

**Keywords:** *O*-GlcNAcylation, *O*-linked N-acetylglucosamine transferase, colon cell lines, colorectal cancer, siRNA

## Abstract

The post-translational modification of proteins by *O*-linked β-N-acetylglucosamine (*O*-GlcNAc) is regulated by a unique couple of enzymes. *O*-GlcNAc transferase (OGT) transfers the GlcNAc residue from UDP-GlcNAc, the final product of the hexosamine biosynthetic pathway (HBP), whereas *O*-GlcNAcase (OGA) removes it. This study and others show that OGT and *O*-GlcNAcylation levels are increased in cancer cell lines. In that context, we studied the effect of OGT silencing in the colon cancer cell lines HT29 and HCT116 and the primary colon cell line CCD841CoN. Herein, we report that OGT silencing diminished proliferation, *in vitro* cell survival and adhesion of primary and cancer cell lines. SiOGT dramatically decreased HT29 and CCD841CoN migration, CCD841CoN harboring high capabilities of migration in Boyden chamber system when compared to HT29 and HCT116. The expression levels of actin and tubulin were unaffected by OGT knockdown but siOGT seemed to disorganize microfilament, microtubule, and vinculin networks in CCD841CoN. While cancer cell lines harbor higher levels of OGT and *O*-GlcNAcylation to fulfill their proliferative and migratory properties, in agreement with their higher consumption of HBP main substrates glucose and glutamine, our data demonstrate that OGT expression is not only necessary for the biological properties of cancer cell lines but also for normal cells.

## Introduction

**Figure 1:**
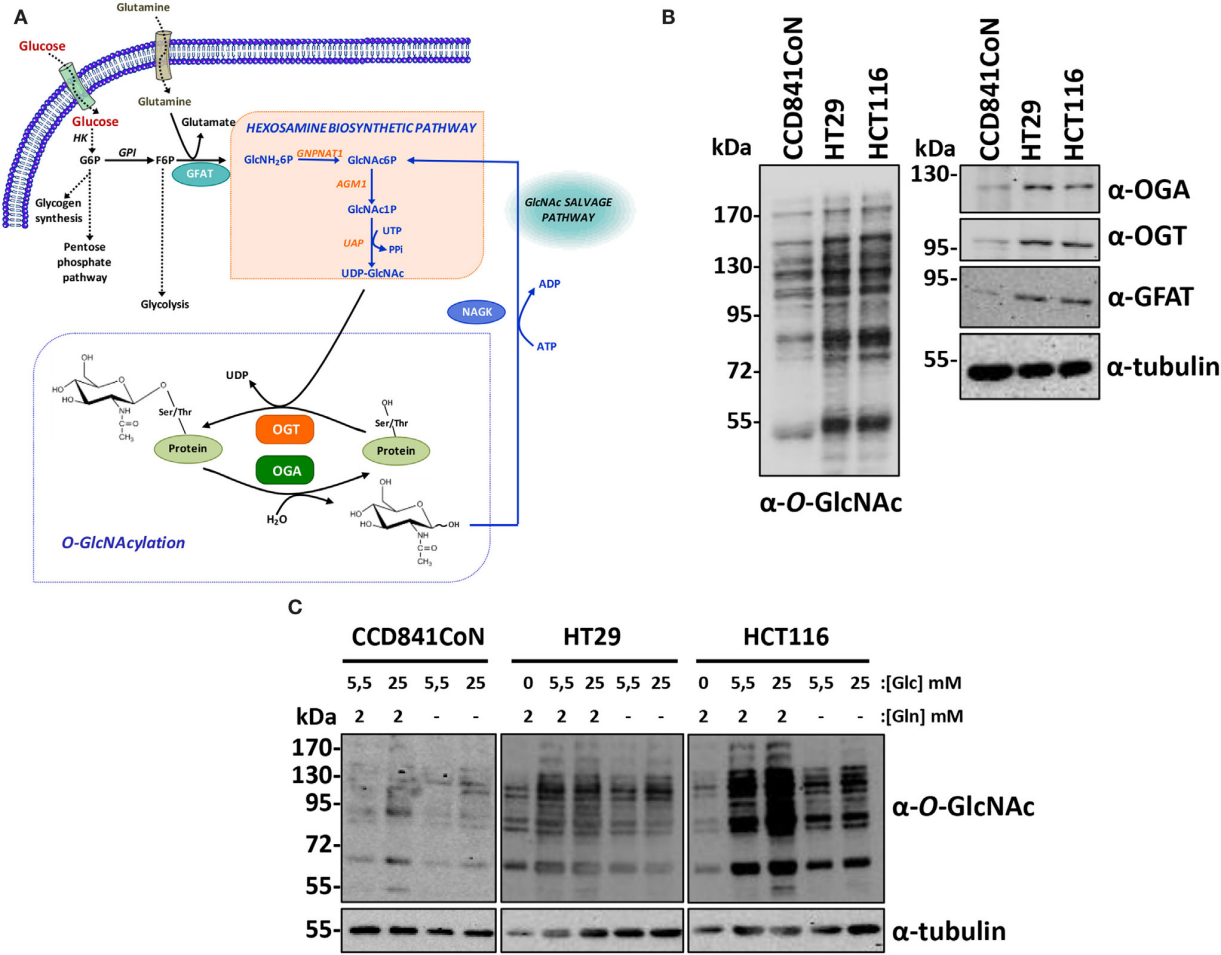
**Colon cancer cell lines have increased-OGT, OGA, and *O*-GlcNAcylated proteins levels**. **(A)** *O*-GlcNAcylation is dependent upon cell nutrient status and is regulated by OGT and OGA. The unique glycosyltransferase OGT transfers the GlcNAc moiety from UDP-GlcNAc to target proteins and the glycosidase OGA hydrolyzes the glycosidic bond; therefore, *O*-GlcNAcylation is versatile. Glucose and glutamine are the main substrates of the hexosamine biosynthetic pathway, which gives rise to UDP-GlcNAc. Moreover, the consumption of these two metabolites is increased in cancer cell lines. The flux of the hexosamine pathway is controlled by the rate-limiting enzyme GFAT. The level of UDP-GlcNAc, and consequently of *O*-GlcNAcylation, is tightly dependent upon the nutrient status of the cell. After its release by OGA, GlcNAc is reactivated by NAGK in the GlcNAc salvage pathway. HK, hexokinase; GPI, phosphoglucose isomerase; GFAT, glutamine:fructose-6-phosphate amidotransferase; GNPNAT1, glucosamine-6-phosphate acetyl transferase; AGM1, phospho-N-acetylglucosamine mutase; UAP, uridine di-phospho-N-acetylglucosamine pyrophosphorylase; NAGK, N-acetylglucosamine kinase; OGA, *O*-GlcNAcase (*O*-linked N-acetylglucosaminidase); OGT, *O*-GlcNAc transferase (*O*-linked N-acetylglucosaminyl transferase). **(B)** *O*-GlcNAcylation, OGT, OGA, and GFAT levels in the three colon cell lines used in the study were assessed by Western blot. **(C)** CCD841CoN, HT29, and HCT116 cells were cultured in various concentrations of glucose (0, 5.5, and 25 mM) and with (2 mM) or without glutamine. *O*-GlcNAcylated proteins were analyzed by Western blot.

*O*-linked β-N-acetylglucosaminylation (*O*-GlcNAcylation) is the modification by a single residue of N-acetylglucosamine (GlcNAc) of nucleocytoplasmic and mitochondrial proteins. This modification is highly dynamic and is regulated by two enzymes: the *O*-GlcNAc transferase (OGT), which adds the residue and the *O*-GlcNAcase (OGA), which removes it (Figure 1A). *O*-GlcNAcylation cycling is involved in many fundamental functions, including translation (1), transcription (2), cell signaling (3), and protein trafficking (4). Deregulation of *O*-GlcNAcylation dynamics actively participates in tumorigenesis and in the etiology of cancer (5). A synergy between unhealthy diet and cancer development is proposed because of the status of OGT’s natural substrate, UDP-GlcNAc, which is positioned at the crossroad of metabolic pathways (6–8). Moreover, glucose transport and consumption is upregulated in cancer cells. This alteration of metabolism is called the “Warburg effect,” Otto Warburg having devoted a large part of his research activities to “the origin of cancer cells” (9).

The initial observation made by Cori and Cori (10) concluded that a hen’s wing having Rous sarcoma produced more lactic acid than the normal wing. Therefore, it was suggested that a deficiency in glucose metabolism was responsible for carcinogenesis even if it is now accepted that it is rather a consequence. Interestingly, cancer cells not only increase glucose consumption but also use more glutamine (11). Glutamine is necessary for both non-essential amino-acids and for purine/pyrimidine base synthesis in highly proliferative cells (11). In cancer cells, glucose is also used for production of amino-acids and lipids. A large part of the increased-glucose flux is diverted toward the pentose phosphate pathway (PPP) to generate deoxyribose needed for the synthesis of DNA. Interestingly, both glutamine and glucose are limiting substrates of the hexosamine biosynthetic pathway (HBP) (12). HBP results in UDP-GlcNAc synthesis subsequently used by OGT to modify proteins (6). Consequently, through *O*-GlcNAcylation, HBP activation is involved in cellular structure construction as well as regulation of metabolic flux, signaling pathways, cell homeostasis processes, and tumorigenesis. Increased *O*-GlcNAcylation levels have been reported in diverse kind of cancers: breast (5, 13, 14), lung (15), liver (16), prostate (17), chronic lymphocytic leukemia (18), colon (15, 19–21), and colitis-associated cancer patients (22). Regarding these recent observations, we wondered whether silencing OGT expression affects biological properties of colon cancer-derived cell lines (HT29 and HCT116) in comparison to a normal colon cell line (CCD841CoN, derived from a fetus). Although *O*-GlcNAcylation and its cycling enzymes are more elevated in cancer cell lines, we found that both normal and cancer cell lines are impacted by siOGT. While the fetal colon cells are highly mobile, we demonstrate that OGT knockdown slows down their migration property in addition to a disturbance of the cytoskeleton. Together, these data show that OGT is crucial for the biological properties of cancer and non-cancer cell lines. However, we suggest that cancer cell lines are highly sensitive to metabolic deprivation and OGT knockdown, accordingly to their high-demand in nutrients, in particular glucose and glutamine.

## Materials and Methods

### Cell Culture and Transfection of siRNA

HT29 and HCT116 cells were maintained in a Dulbecco’s modified Eagle’s medium (DMEM) and CCD841CoN cells were maintained in an Eagle’s Minimum Essential Medium (EMEM) (Lonza) (Table 1). The three cell lines were maintained in a medium supplemented with 10% (v/v) fetal calf serum, 2 mM l-glutamine, 5 IU/mL penicillin, and 50 μg mL^−1^ streptomycin at 37°C in a 5% (v/v) CO_2_-enriched humidified atmosphere. Cells were reverse-transfected with Lipofectamine RNAiMax (Life Technologies) according to manufacturer’s instructions using 5 nM small interfering RNA targeting OGT (21) or a control siRNA (MISSION siRNA universal negative control #1, SIGMA).

**Table 1:** **Characteristics of the cell lines used in the study**.

Cell line	Gender, age, disease, MSI status	Characterized deletions, mutations, and comments
CCD841CoN	Female, 21-week gestation (fetus), normal colon	According to ATCC, *the definitive evidence of epithelial origin is lacking*
HT29	Female, 44 years, colorectal adenocarcinoma, MSS	Deletion in APC (del1555_2843)
		Mutation in p53 (codon 273: R273H)
		Mutation in BRAF (codon 600: V600E)
		Mutation in PI3KCA (P449T)
HCT116	Male, 48 years, colorectal carcinoma (proximal), HNPCC, MSI	Mutation in Ras (codon 13: G13D)
		Mutation in PI3KCA (codon 1047: H1047R)
		Deletion in β-catenin (del45)

### SDS-PAGE, Western Blotting, and Antibody Staining

Equal amounts of protein (determined by the BCA method) were resolved on 10% SDS-PAGE under reducing conditions and proteins were electroblotted on nitrocellulose (GE Healthcare). Efficiency of the transfer and equal loading were verified using Ponceau red staining. Membranes were first saturated for 45 min with 5% (m/v) non-fatty acid milk in Tris-buffered saline (TBS)-Tween buffer [15 mM Tris/HCl, 140 mM NaCl, and 0.05% Tween20 (v/v), pH 8.0]. Mouse monoclonal anti-*O*-GlcNAc (RL2, Ozyme) was used at a dilution of 1:4,000. Chicken anti-OGA (345, generously provided by Hart, the Johns Hopkins School of Medicine, Baltimore, MD, USA) was used at a dilution of 1:2,000. Mouse monoclonal anti-tubulin (Santa Cruz Biotechnology) and mouse polyclonal anti-GAPDH (Santa Cruz Biotechnology) were used at a dilution of 1:3,000; rabbit polyclonal anti-OGT (TI14, Sigma-Aldrich) was used at a dilution of 1:4,000; anti-GFAT (Abcam). Membranes were incubated with the different antibodies overnight at 4°C, then washed three times with TBS-Tween for 10 min, and incubated with either an anti-rabbit or an anti-mouse horseradish peroxidase-labeled secondary antibody at a dilution of 1:10,000 for 1 h. Finally, three washes of 10 min each were performed with TBS-Tween and the detection was carried out by chemiluminescence imaging (Fusion Solo system, Vilber Lourmat).

### Cell Adhesion Assay

Cells (1 × 10^4^) were seeded 48 h after transfection with siRNA targeting OGT or a control siRNA in 96-well plates (Thermo Fisher Scientific) in DMEM for HCT116 and HT29, and EMEM for CCD841 CoN, containing 10% (v/v) fetal calf serum. At the indicated time periods, medium containing not attached cells was removed and replaced with fresh medium. Viability of adherent cells was analyzed using the MTS method (Promega) according to the manufacturer’s instructions. Cell adhesion was measured in three independent experiments (*n* = 8).

### Proliferation Assays

HCT116 and HT29 cells (2 × 10^3^) and CCD841 CoN (1 × 10^3^) cells were seeded in 96-well plates (Thermo Fisher Scientific) and grown in DMEM (for HCT116 and HT29) or EMEM (for CCD841CoN) medium containing 10% (v/v) fetal calf serum. Cells were transfected two times with siRNA targeting OGT or a control siRNA. Cell growth was analyzed for the indicated time periods using the MTS reagent (Promega) according to the manufacturer’s instructions. Cell proliferation was evaluated in three independent experiments (*n* = 8).

### *In Vitro* Cell Survival Assays

Cells (2 × 10^3^) were seeded in 100-mm Petri dishes. After attachment, cells were transfected four times with siRNA targeting OGT or a control siRNA. Two weeks later, cells were fixed in 4% (m/v) paraformaldehyde (Sigma-Aldrich) for 20 min at room temperature and stained with crystal violet (0.1% w/v, 30 min, at room temperature) for 30 min at room temperature. Colonies were then washed several times with water and left to dry at room temperature. *In vitro* cell survival assays were performed in two independent experiments (*n* = 4).

### Cell Migration Analysis

#### Wound Healing Assays

Wound healing assays were performed using culture-insert-μDish (Ibidi) composed of two chambers (growth area per well 0.22 cm^2^) separated by a wall (width of 500 μm). Culture-inserts were put in 12-well plates. 1 × 10^4^ CCD841CoN and 4 × 10^4^ HCT116 and HT29 cells were seeded into the chambers 48 h after transfection. After cell attachment overnight at 37°C in 10% (v/v) fetal calf serum-containing medium (DMEM for HCT116 and HT29 and EMEM for CCD841CoN) culture-inserts were gently removed to form the cell-free gap. For each cell line, pictures were taken 24 h later to monitor the healing of the cell-free gap on a Nikon Eclipse Ti-U microscope. Wound healing assays were performed in three independent experiments (*n* = 6).

#### Transwell System

Forty-eight hours after transfection cells (5 × 10^4^) were seeded in the upper surface of a Transwell 12-well (size of the pores, 8 μm) (BD Biosciences) and cultured for 24 h in a 0.2% (v/v) fetal calf serum-containing medium (DMEM for HCT116 and HT29 and EMEM for CCD841 CoN). After incubation, cells were fixed in 4% (m/v) paraformaldehyde for 20 min at room temperature and stained with Hoechst 33258 for 15 min in the dark at room temperature. Then, cells in the upper chamber of the well’s porous membrane were removed by scraping with a cotton swab and washed several times with phosphate-buffered saline (PBS, pH 7.2). The membrane was mounted on slide with Glycergel mounting medium (Dako). The migrated cells were counted under ×20 magnification and the mean number of cells was evaluated in three independent experiments (*n* = 6).

### Confocal Microscopy

Cells were cultured on glass coverslips in DMEM (for HCT116 and HT29) and EMEM (for CCD841CoN) medium containing 10% (v/v) fetal calf serum. Cells were washed twice in PBS and fixed in 4% (m/v) paraformaldehyde for 20 min at room temperature. After washing with PBS, cells were blocked and permeabilized in PBS containing 0.2% (m/v) BSA and 0.1% (m/v) saponin for 1 h. Cells were incubated for 4 h at room temperature with polyclonal anti-tubulin (1:200) (Santa Cruz Biotechnology) or monoclonal anti-vinculin (1:200) (Abcam), diluted in PBS-BSA-saponin. Cells were washed three times in PBS–BSA–saponin and incubated with Alexa Fluor^®^ 488 and Alexa Fluor^®^ 568 (Life Technologies) or CytoPainter Phalloidin-iFluor 488 Reagent (1:1000) (Abcam) in PBS–BSA–saponin for 1 h at room temperature. Following washings, cells were stained with Hoechst during 15 min in the dark at room temperature. After washings with PBS and deionized water, cells were mounted with Glycergel mounting medium. Data were finally collected using the ZEN2012 software.

### Statistical Analysis

Student’s *t*-test (Excel) was used for statistical analysis (unpaired/two-tailed); *p*-values were calculated and reported accordingly (*P < 0.05, **P < 0.01, ***P < 0.001).

## Results

### HT29 and HCT116 Cells Exhibit Higher Levels of *O*-GlcNAcylation Cycling Enzymes Compared to CCD841CoN

Two colon cancer cell lines, HT29 and HCT116, respectively, derived from an adenocarcinoma and from a carcinoma (Table 1) and the fetal colon cell line CCD841CoN were analyzed by Western blot to determine to the levels of *O*-GlcNAcylation, OGT, OGA, and Glutamine:Fructose-6-P amidotransferase (GFAT), the rate-limiting enzyme of the HBP (Figures 1A,B). As shown by western blotting results, *O*-GlcNAcylated proteins levels, as well as OGA, OGT, and GFAT protein expression were much more elevated in cancer cell lines compared to the fetal cell line (Figure 1B), in accordance with our previous study (23). We next checked whether the three cell lines’ *O*-GlcNAcylation levels were dependent upon glucose and glutamine, the two pivotal substrates of the HBP (Figure 1A). Thus, overnight depletion of either glucose or glutamine reduced *O*-GlcNAcylation processes, more particularly in HT29 and HCT116 cells (Figure 1C), which are high consumers of these two nutrients like most cancer cell lines (7–11). Moreover, we noticed that CCD841CoN were particularly sensitive to glucose starvation, leading to a high mortality rate.

Therefore, we next assessed the impact of OGT knockdown on the biological properties of these cell lines.

### OGT Knockdown Decreases Cell Proliferation, Reduces *In Vitro* Cell Survival, and Impairs Cell Adhesion

**Figure 2:**
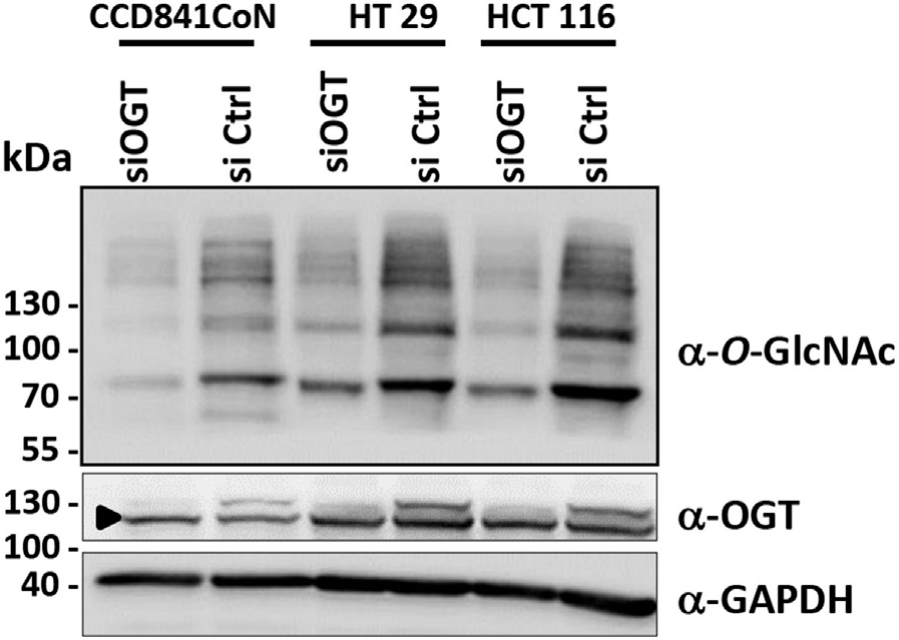
**OGT knockdown decreases *O*-GlcNAcylation contents in colon cells**. Forty-eight hours after transfection, the efficiency of siOGT vs. siCtrl was tested in the three cell lines used in the study. OGT and *O*-GlcNAcylation levels were measured by Western blot. The arrowhead indicates an unspecific band.

The efficiency of siOGT was checked for the three cell lines. As expected, transfection of HCT116, HT29, and CCD841CoN with siOGT decreased the expression of the glycosyltransferase and drastically reduced *O*-GlcNAcylation levels (Figure 2).

**Figure 3:**
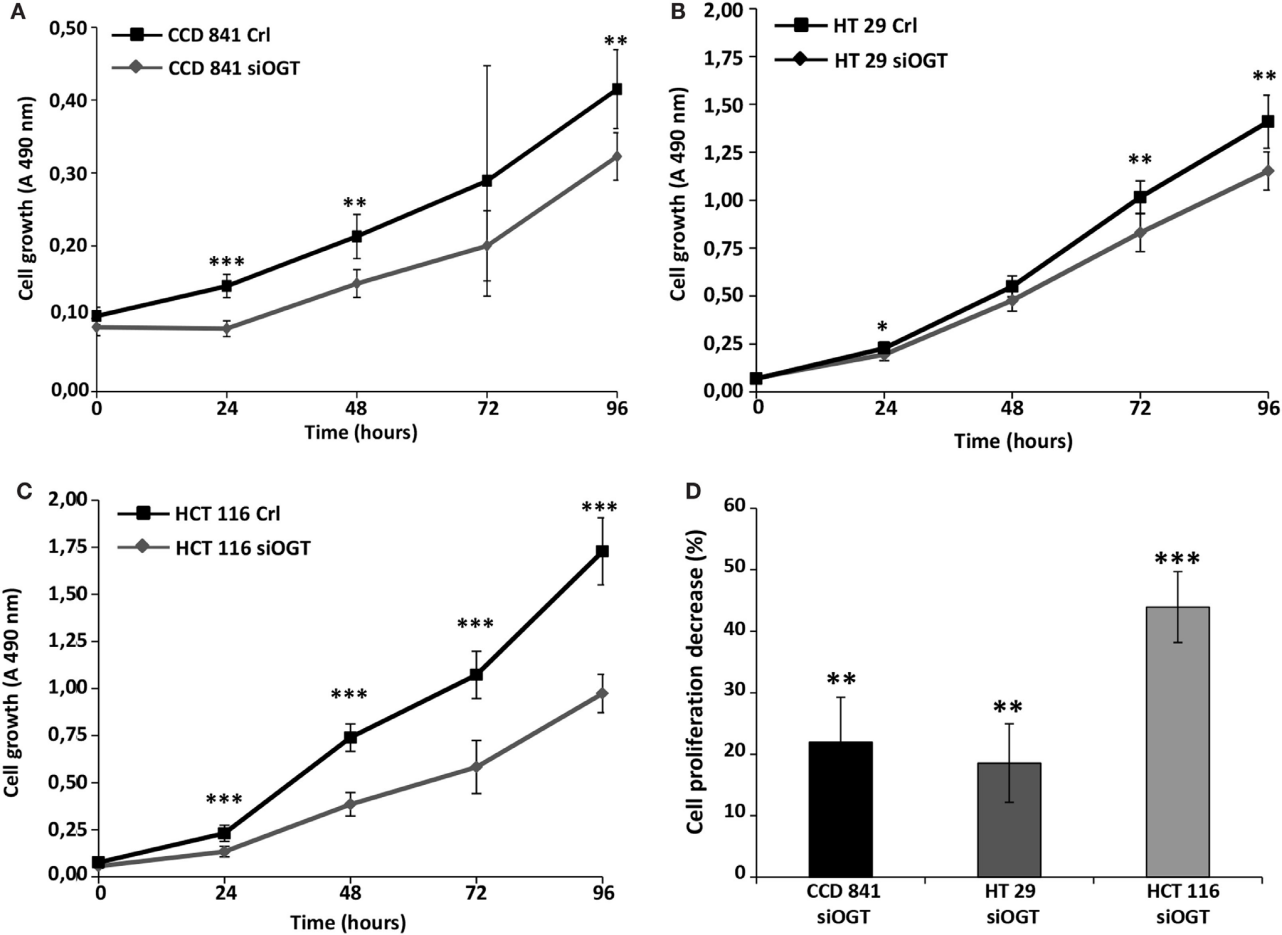
**OGT silencing slows down cell proliferation**. Colon cell proliferation was monitored for 4 days in conditions of OGT silencing vs. siCtrl. Each day, cell growth was determined using the MTS reagent method. **(A)** CCD841CoN; **(B)** HT29; **(C)** HCT116. **(D)** The decrease of cell proliferation for each cell line was calculated and reported (±SD). (Student’s *t*-test: *P < 0.05, **P < 0.01, ***P < 0.001).

**Figure 4:**
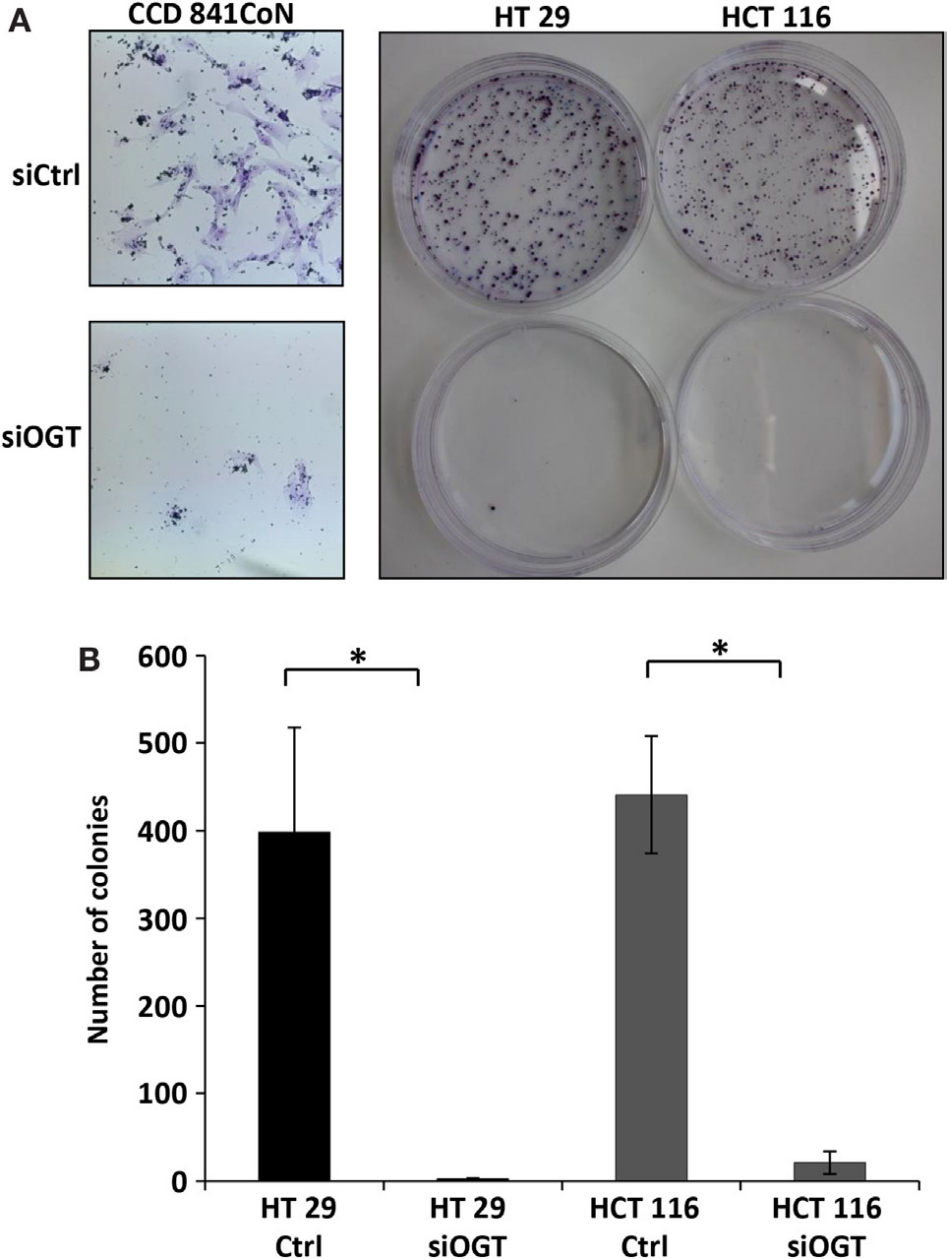
**OGT knockdown reduces colony forming potential of colon cells**. CCD841CoN, HT29, and HCT116 cells were seeded and transfected with siCtrl or siOGT in 100-mm Petri dishes. Colonies were stained with crystal violet after 2 weeks. **(A)** Representative results of colony staining in siCtrl and siOGT-transfected CCD841CoN, HT29, and HCT116 cells. For CCD841CoN cell line pictures were taken under ×20 magnification. **(B)** Colony counting in siCtrl and siOGT for HT29 and HCT116 cells were quantified (mean values ± SD). (Student’s *t*-test: *P < 0.05).

We previously observed that the inhibitor of OGA, NButGT (24), accelerated the proliferation rate of MCF7 cells (23), whereas the potent OGT inhibitor, Ac-5SGlcNAc (25), slightly delayed cell proliferation (26). In the present study, we tested the impact of siOGT on the proliferation rate of CCD841CoN, HT29, and HCT116 cells (Figures 3A–C, respectively). As expected and in agreement with our previous observations (26), OGT silencing decelerated the proliferation rate of the three cell lines, with an average 20% decrease for CCD841CoN and HT29, and 45% decrease for HCT116 (Figure 3D). To go further, we tested the ability of colon cells to grow into a colony (*in vitro* cell survival assay) in response to siOGT (Figure 4A). Both for HT29 and HCT116 cells, the reduction of OGT expression dramatically decreased *in vitro* survival compared with siCtrl-transfected cells (Figure 4B). The ability of a single cell to grow into a colony is characteristic of cancer cells. Accordingly, we were unable to assess the formation of colonies for the primary cell line CCD841CoN. These experiments showed that OGT and, consequently, *O*-GlcNAcylation are needed for colon cells growth.

**Figure 5:**
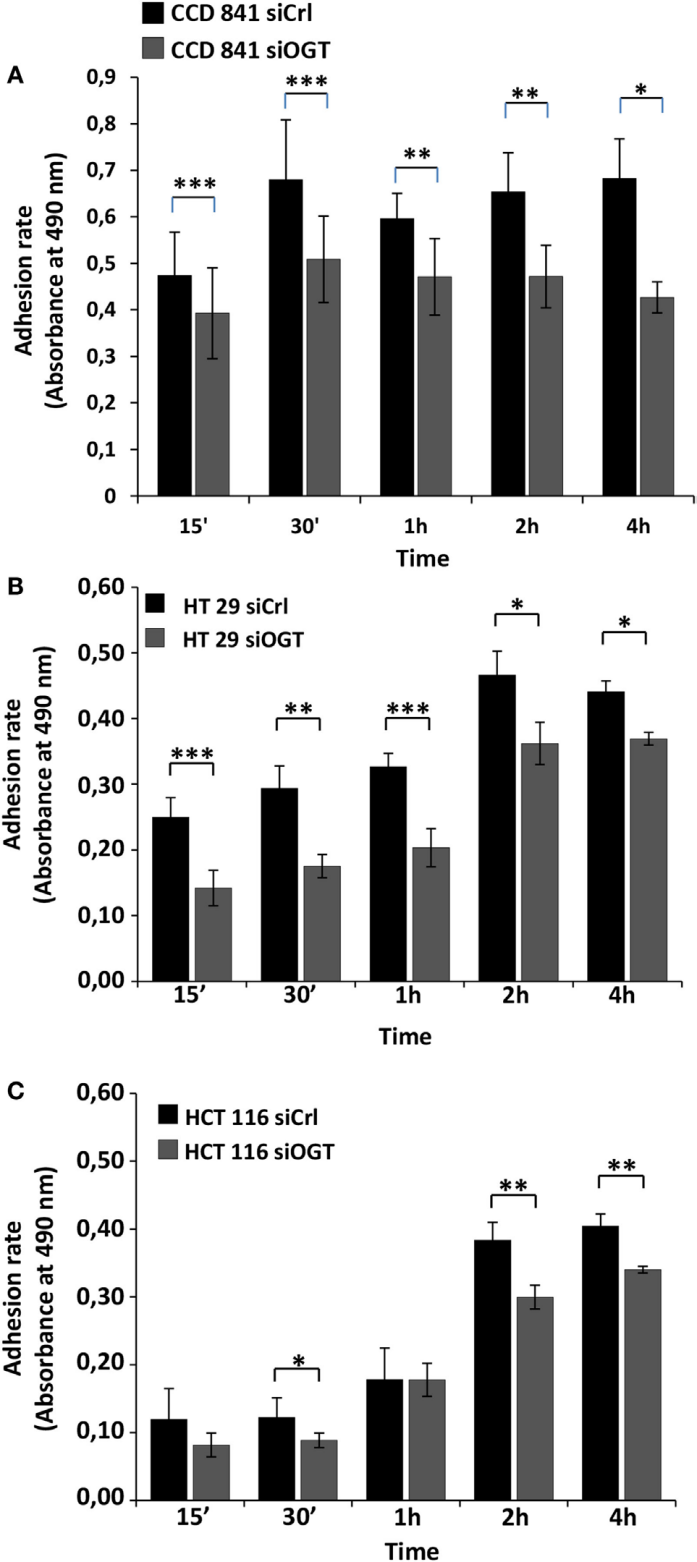
**OGT silencing reduces cell adhesion**. Cell adhesion was monitored for the different cell lines 48 h after siOGT or siCtrl treatment from 15 min to 4 h after seeding as detailed in the experimental procedures section. Mean values (±SD) were reported. **(A)** CCD841CoN; **(B)** HT29; **(C)** HCT116. (Student’s *t*-test: *P < 0.05, **P < 0.01, ****P < 0.001).

We next performed cell adhesion assays for the indicated time periods (Figure 5). While siOGT decreased the adhesion of the three cell lines, we observed a higher impact of OGT silencing on the adhesion of HT29 and CCD841CoN cells. OGT is, therefore, necessary for adhesion of colon cells.

### OGT Knockdown Reduces Migration of Colon Cells

**Figure 6:**
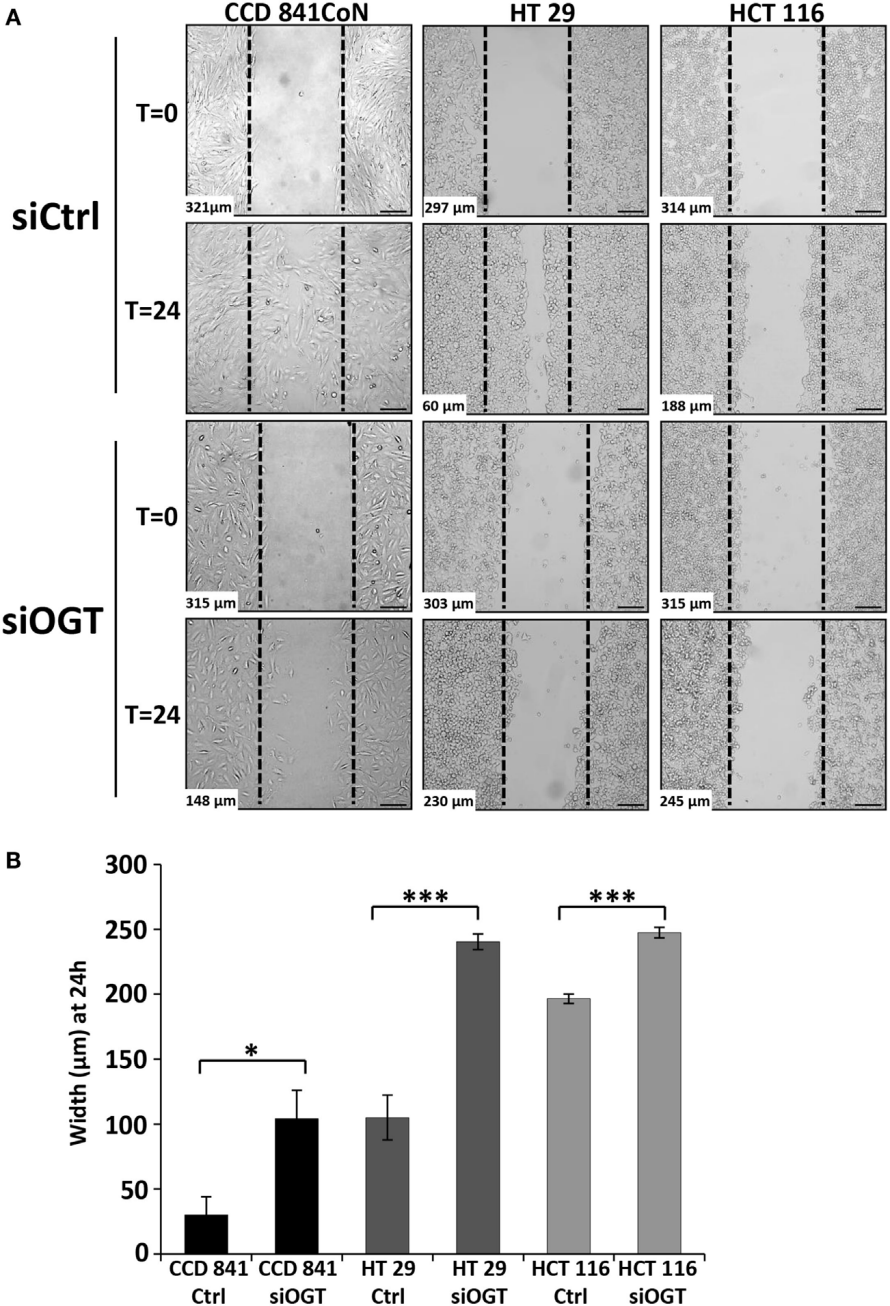
**OGT silencing slows down colon cells migration**. **(A)** Representative wound healing assays of siCtrl and siOGT-transfected CCD841CoN, HT29, and HCT116 cells are represented. The dotted lines correspond to the wound at *t* = 0. At the bottom left is indicated the width of the wound at *t* = 0 or at *t* = 24 h established by image analysis. Bar, 100 μm **(B)** Mean values (±SD) of the width of the wounds at *t* = 24 h in siCtrl and siOGT for the three colon cell lines. (Student’s *t*-test: *P < 0.05, ***P < 0.001).

**Figure 7:**
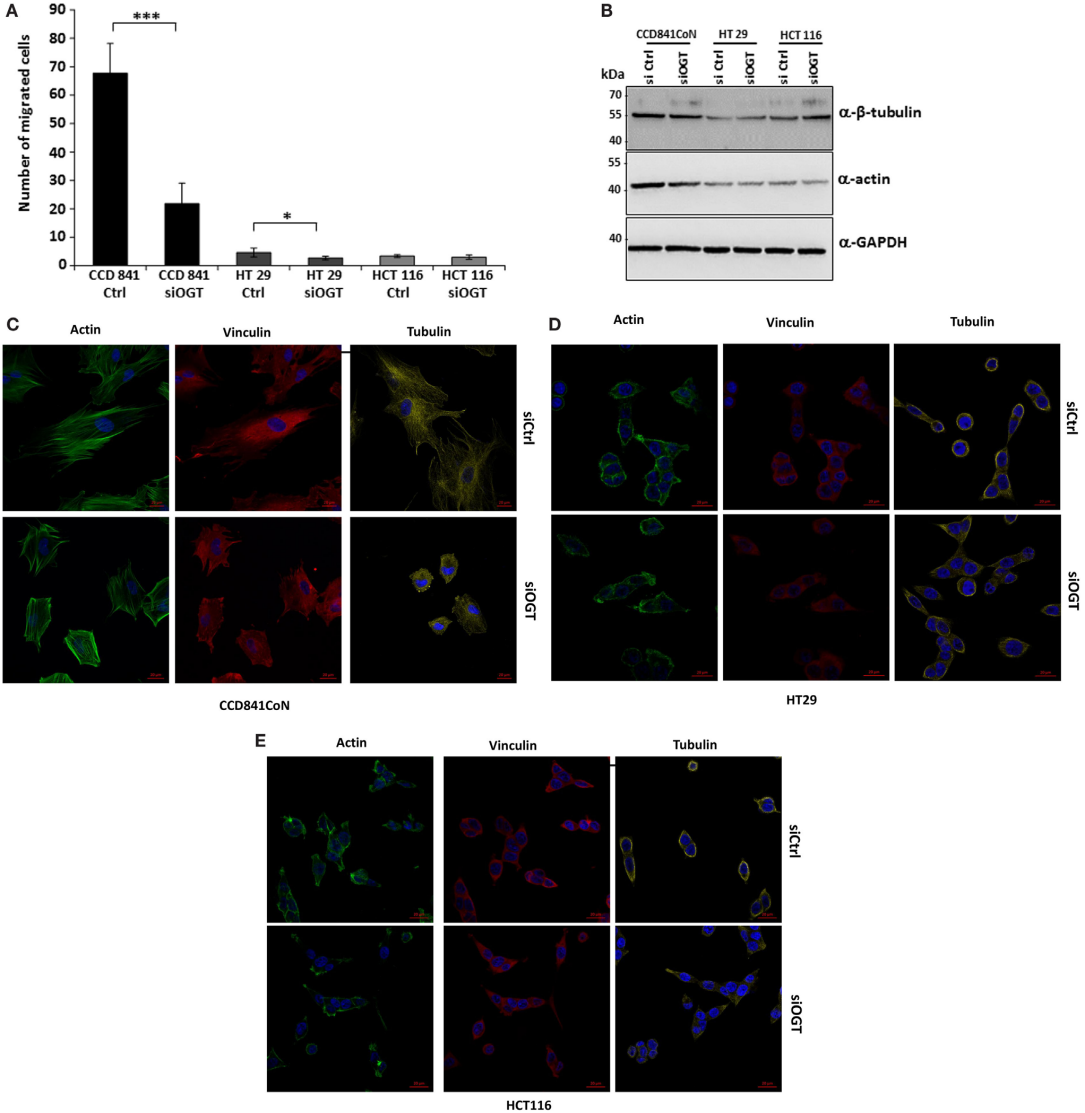
**OGT silencing reduces migration of CCD841CoN and HT29 cells in Boyden chamber system**. **(A)** Colon cells were transfected with siCtrl or siOGT. Twenty-four hours after seeding in Transwell 12-well plates, cells were visualized by Hoechst staining. Mean values (±SD) of the migrated cells are reported. **(B)** Expression of β-tubulin and actin were analyzed by Western blot for siCtrl vs. siOGT-transfected colon cell lines. GAPDH was used as a loading control. **(C–E)** Morphology of CCD841CoN **(C)**, HT29 **(D)**, and HCT116 **(E)** cells was assessed by confocal microscopy. Tubulin and vinculin were visualized using specific-directed antibody and actin using the CytoPainter Phalloidin-iFluor 488 Reagent. Bar, 20 μm.

We wondered whether OGT is involved in the migration of colon cells. For this matter, we used two distinct approaches: the wound healing assay using culture-inserts μDish and the Transwell system assay, a more quantitative method. Wound healing was assessed 24 h after the culture-inserts were removed (Figure 6). While CCD841CoN were able to fill in the cell-free gap in 24 h, the gap separating the two edges of the migratory HT29 cells was tight at *t* = 24 h. However, HCT116 only slowly recovered the wound after 24 h. OGT knockdown impaired the closure of the wound for CCD841CoN and HT29 cells as no significant migration was measured after siOGT treatment in comparison to siControl. Nevertheless, even if the migration of the HCT116 cells was slow, siOGT also delayed significantly this process (Figure 6B). Furthermore, these first observations were corroborated by the more quantitative Transwell assay (Figure 7A). HCT116 cells did not display any significant migration across the filter pores even in the siControl conditions. Regarding HT29, while these cells harbored a weak migration in Boyden chambers, a significant decrease was measured when OGT was silenced. Surprisingly, despite their size, CCD841CoN cells exhibited a high capacity of migration compared to HT29 and HCT116 in Boyden chambers. OGT silencing dramatically reduced their migratory capabilities, in accordance with wound healing assays.

We hypothesized that a larger cytoskeleton network could confer the high-migratory properties of CCD841CoN cells of which the epithelial origin has not been clearly established (Table 1). First, we showed that actin, the major component of the microfilament network, was more heavily expressed in CCD841CoN cells compared to HT29 and HCT116 cells (Figure 7B). Interestingly, siOGT did not impact the expression of both actin and tubulin, another major component of the cytoskeleton network. Analysis of the cytoskeleton by confocal microscopy (Figures 7C–E) indicated that the cytoskeletal networks are more elaborated in CCD841CoN, which reinforces the doubt about the epithelial origin of this cell line according to ATCC. Surprisingly, OGT silencing did not impact the expression of actin, tubulin, and vinculin, a cytoskeletal protein associated with cell–cell and cell–matrix junctions. On the other hand, siOGT greatly affected the cytoskeletal networks and cell morphology in CCD841CoN cells. The cell shape appeared stocky and stunted, whereas the microfilament network, responsible for cell migration, was less extended.

## Discussion

*O*-GlcNAcylation is fundamental for cell viability as demonstrated by the non-viability of OGT-depleted ES cells (27). Whereas three isoforms were described for OGT (nucleocytoplasmic, short, and mitochondrial isoforms), this enzyme is encoded by a single gene and downregulating its expression or inhibiting its catalytic activity affects major fundamental biological functions (3, 26, 27). Moreover, a deregulation in the *O*-GlcNAcylation cycling is involved in the etiology of diverse pathologies, such as type-2 diabetes, Alzheimer’s, and cancers (6). Intriguingly, due to the correlation between UDP-GlcNAc, *O*-GlcNAcylation, and nutrients availability, diseases associated with unhealthy diet and metabolic disorders may be tightly linked to *O*-GlcNAcylation imbalance (28). It is, therefore, crucial to better understand through which metabolic mechanisms lifestyles negatively impact human health. Particularly, some cancers, among which colorectal cancers, are synergistically favored by metabolic problems and unhealthy lifestyles (29, 30). Elevated-*O*-GlcNAcylation level was described in many cancers, even if the reasons and impacts of this increased level remain obscure. For example, breast cancer cells have increased-*O*-GlcNAcylation and OGT levels, and a reduction of OGT expression correlated to tumor growth decrease (5). High levels of OGT and its product, *O*-GlcNAcylation, were also reported in colon cancers and cancer cell lines (15, 19–21) and colitis-associated cancer patients (22) but very few initiatives to measure the effect of OGT knockdown were conducted. Among those few studies, OGA inhibition or shOGT exhibited no significant effect on HT29 cell invasion but enhanced and reduced, respectively, their anchorage-independent growth (15). Furthermore, OGT knockdown inhibited anchorage-independent growth (14), but contradictory results were also reported (19). This last study indicated that OGA silencing changed the anchorage-independent growth and the morphology of the primary colorectal cancer cell SW480 and of its metastatic clone SW620 (19). Whereas one previous study showed altered gene expression related to actin reorganization and cell migration in siOGA-transfected cells (19), we found that OGT silencing also modifies cell shape features of the fetal human colon cell line CCD841CoN. In our hands, siOGT did not modify the expression of actin but we suspected that actin-binding proteins are up- or downregulated when OGT is silenced. In that sense, OGT promotes breast cancer cells invasion in a cofilin-dependent manner. *O*-GlcNAcylation of cofilin at Ser108 localizes this actin-interacting protein to invadopodia (31). Also, under conditions of low OGT expression, actin may be itself affected by defective *O*-GlcNAcylation. In a previous work, we mapped a major *O*-GlcNAcylation site within the 318–324 region of β-actin expressed in *Xenopus laevis* oocytes (32) and later, one *O*-GlcNAcylation site was localized in the domain four of rat actin (33). Nevertheless, the function of actin *O*-GlcNAcylation remains to be established. In parallel, *O*-GlcNAcylation was also widely studied on tubulin, another major component of the cytoskeleton network. *O*-GlcNAcylation of α-tubulin reduces heterodimerization of α/β-tubulins and *O*-GlcNAcylated forms of tubulins are unable to polymerize into microtubule (34). Moreover, α-tubulin is heavily *O*-GlcNAcylated in primary colorectal cancer (20). These two independent studies tend to support our observations of a disorganization of microtubules in the primary colon cell line, while no significant effect of siOGT was found in the two colon cancer lines.

Beyond the effect of *O*-GlcNAcylation on structural proteins in a pathologic context, downregulation of OGT must interfere with the expression and/or the activity of regulatory proteins. A comparison between primary breast malignant tumors and benign tumors revealed the *O*-GlcNAcylation of crucial components of the “Warburg effect” only in cancer (14). One of the characteristics of cancer cells is the shift from an oxidative to a non-oxidative consumption of glucose. Oncogenic signaling pathways controlling the transcription factor hypoxia-inducible factor-1 (HIF1) alpha are responsible for this metabolic shift.

HIF1α stability is dependent upon *O*-GlcNAcylation level (35). GLUT1 expression, one of the HIF1α’s target genes, is more heavily expressed when OGT is activated. Consequently, glucose transport into the cell is increased. Most of the glycolytic enzymes are modified by *O*-GlcNAcylation (32, 36, 37). Among those, the enzyme phosphofructokinase-1 (PFK1) (38) controls the entry of glucose into glycolysis. *O*-GlcNAcylation of PFK1 at Ser509 prevents the binding of the activator Fru-2,6-bis-phosphate. Consequently, this modification diverts the use of glucose to the PPP to produce pentoses and NADPH_2_, respectively, used for nucleic acids and lipids biosynthesis. This confers an advantage for cancer cell to increase their proliferation rate.

*O*-GlcNAc transferase is critical for normal cells and cancer cell homeostasis and adaptation to environment. Due to the plethora of OGT’s targets, it is difficult to assign precisely the impact of OGT silencing. In light of the different elements exposed above, we suggest that a default of *O*-GlcNAcylation impacts on cell architecture as attested by the alteration of morphology observed in CCD841CoN cells and of metabolic routes. Moreover, knocking-down OGT also results in inactivation of mitogen signaling pathways as previously established (3, 26, 39, 40).

Our observations indicate that OGT is crucial for the biological properties of normal colon-derived cells and colon cancer cell lines. However, colon cancer cells express higher amounts of OGT and *O*-GlcNAcylation than normal cells. Due to the addiction of cancer cells for glutamine and glucose (7–10), the main substrates of HBP, it could be suspected that cancer cells were much more sensitive to changes in *O*-GlcNAcylation levels than normal cells while we found that both colon cancer and primary cell lines were affected by OGT silencing. OGT also interferes with cell migration, especially for the fetal cell line CCD841CoN, by reducing the size of the actin network, which participates in the alteration of the cell morphology. Along this study, we demonstrated that OGT impacts proliferation and migration of normal as well as cancer colon cells. This work highlights how *O*-GlcNAcylation, through the modulation of basic biological features, controls the properties of primary cells and also might initiate nutrient-responsive cancers, such as colorectal cancer.

## Author Contributions

AS designed, performed, and analyzed the experiments, and wrote the experimental procedures. SS and VD initiated the project, conceived, and performed experiments. SS edited the manuscript. SB and R-AT performed and analyzed the experiments. XB analyzed the experiments and managed the study at INSERM U908. IY-B designed, performed, and analyzed the experiments. TL initiated the project, conceived, and coordinated the study, and wrote the paper. All authors reviewed the results and approved the final version of the manuscript.

## Conflict of Interest Statement

The authors declare that the research was conducted in the absence of any commercial or financial relationships that could be construed as a potential conflict of interest.

## Abbreviations

GFAT, Glutamine:Fructose-6-phosphate amidotransferase; NAGK, N-acetylglucosamine kinase; OGA, *O*-GlcNAcase (*O*-linked N-acetylglucosaminidase); OGT, *O*-GlcNAc transferase (*O*-linked N-acetylglucosaminyl transferase).
